# Supplementary material for: Resolving the full spectrum of human genome variation using Linked-Reads
Source: Genome Res. 2019 Apr;29(4):635–45. doi: 10.1101/gr.234443.118 (PMC6442396; doi:10.1101/gr.234443.118)
Supplement: Supplemental Material [file supp_gr.234443.118_Supplemental_longranger-2.2.zip › longranger-2.2.tar.bz2!/lr2.2-public/lib/stan/cmdstan/stan/doc/api/html/structstan_1_1gm_1_1validate__sample.html]

Stan: stan::gm::validate\_sample Struct Reference


|  |  |
| --- | --- |
|  | Stan  2.5.0  probability, sampling & optimization |


- Main Page
- Related Pages
- Namespaces
- Classes
- Files

- Class List
- Class Index
- Class Hierarchy
- Class Members

All Classes Namespaces Files Functions Variables Typedefs Enumerations Enumerator Friends Macros Pages

- stan
- gm
- validate\_sample

Classes |
Public Member Functions |
Static Public Member Functions |
List of all members

stan::gm::validate\_sample Struct Reference

`#include <statement_grammar_def.hpp>`

|  |  |
| --- | --- |
| Classes | |
| struct | result |
|  | |

|  |  |
| --- | --- |
| Public Member Functions | |
| bool | is\_double\_return (const std::string &function\_name, const std::vector< expr\_type > &arg\_types, std::ostream &error\_msgs) const |
|  | |
| bool | operator() (const sample &s, const variable\_map &var\_map, std::ostream &error\_msgs) const |
|  | |

|  |  |
| --- | --- |
| Static Public Member Functions | |
| static bool | is\_univariate (const expr\_type &et) |
|  | |

## Detailed Description

Definition at line 219 of file statement\_grammar\_def.hpp.

## Member Function Documentation

|  |  |  |  |
| --- | --- | --- | --- |
| bool stan::gm::validate\_sample::is\_double\_return | ( | const std::string & | *function\_name*, |
|  |  | const std::vector< expr\_type > & | *arg\_types*, |
|  |  | std::ostream & | *error\_msgs* |
|  | ) |  | const |

inline

Definition at line 223 of file statement\_grammar\_def.hpp.

|  |  |  |  |  |  |
| --- | --- | --- | --- | --- | --- |
| static bool stan::gm::validate\_sample::is\_univariate | ( | const expr\_type & | *et* | ) |  |

inlinestatic

Definition at line 230 of file statement\_grammar\_def.hpp.

|  |  |  |  |
| --- | --- | --- | --- |
| bool stan::gm::validate\_sample::operator() | ( | const sample & | *s*, |
|  |  | const variable\_map & | *var\_map*, |
|  |  | std::ostream & | *error\_msgs* |
|  | ) |  | const |

inline

Definition at line 235 of file statement\_grammar\_def.hpp.

---

The documentation for this struct was generated from the following file:

- src/stan/gm/grammars/statement\_grammar\_def.hpp


---

[ Stan Home Page ]

*© 2011–2014,
Stan Development Team.*
